# Simultaneous PET/MRI: The future gold standard for characterizing motor neuron disease—A clinico-radiological and neuroscientific perspective

**DOI:** 10.3389/fneur.2022.890425

**Published:** 2022-08-17

**Authors:** Freimut D. Juengling, Frank Wuest, Sanjay Kalra, Federica Agosta, Ralf Schirrmacher, Alexander Thiel, Wolfgang Thaiss, Hans-Peter Müller, Jan Kassubek

**Affiliations:** ^1^Division of Oncologic Imaging, University of Alberta, Edmonton, AB, Canada; ^2^Neuroscience and Mental Health Institute, University of Alberta, Edmonton, AB, Canada; ^3^Faculty of Medicine, University Bern, Bern, Switzerland; ^4^Department of Neurology, University of Alberta, Edmonton, AB, Canada; ^5^Division of Neuroscience, San Raffaele Scientific Institute, University Vita Salute San Raffaele, Milan, Italy; ^6^Medical Isotope and Cyclotron Facility, University of Alberta, Edmonton, AB, Canada; ^7^Lady Davis Institute for Medical Research, Department of Neurology and Neurosurgery, McGill University, Montreal, QC, Canada; ^8^Department of Nuclear Medicine, University of Ulm Medical Center, Ulm, Germany; ^9^Department of Diagnostic and Interventional Radiology, University of Ulm Medical Center, Ulm, Germany; ^10^Department of Neurology, Ulm University Medical Center, Ulm, Germany

**Keywords:** ALS, motor neuron disease, PET/MRI, DTI, fMRI, SV2A, TrkB/BDNF

## Abstract

Neuroimaging assessment of motor neuron disease has turned into a cornerstone of its clinical workup. Amyotrophic lateral sclerosis (ALS), as a paradigmatic motor neuron disease, has been extensively studied by advanced neuroimaging methods, including molecular imaging by MRI and PET, furthering finer and more specific details of the cascade of ALS neurodegeneration and symptoms, facilitated by multicentric studies implementing novel methodologies. With an increase in multimodal neuroimaging data on ALS and an exponential improvement in neuroimaging technology, the need for harmonization of protocols and integration of their respective findings into a consistent model becomes mandatory. Integration of multimodal data into a model of a continuing cascade of functional loss also calls for the best attempt to correlate the different molecular imaging measurements as performed at the shortest inter-modality time intervals possible. As outlined in this perspective article, simultaneous PET/MRI, nowadays available at many neuroimaging research sites, offers the perspective of a one-stop shop for reproducible imaging biomarkers on neuronal damage and has the potential to become the new gold standard for characterizing motor neuron disease from the clinico-radiological and neuroscientific perspectives.

## Introduction

Based on current guidelines, clinical workup of amyotrophic lateral sclerosis (ALS) will include neuroimaging to rule out structural lesions and neurologic conditions that sometimes account for early clinical features seen in patients suspected of having primary motor neuron disease (1). The clinical management of this highly invalidating condition, however, clearly necessitates, as early as possible, accurate diagnostic and prognostic information on the associated motor neuron degeneration to direct appropriate clinical handling of the individual patient. Neuroimaging has proven to provide reliable *in vivo* biomarkers to better define the various clinical entities within ALS and to provide additional complementary information to the standard clinical workup (2).

## Current situation of neuroimaging for motor neuron disease

### Instrumentation and image processing—MRI

Recent MRI applications for motor neuron disease including ALS have focused both on quantitative and qualitative analysis of structural changes in T1-weighted images as assessed by automatic analysis approaches and, in a body of more recent studies, on the analysis of microstructural alterations within the brain and spinal cord by the application of varieties of diffusion-weighted MRI sequences.

Additionally, substantial efforts have been made utilizing PET to generate newer and more biologically based classifications of ALS and its subtypes (3–5). PET, as a non-invasive *in vivo* imaging technique, provides quantitative data at the molecular level, with novel radiotracers targeting neurons, microglia and astrocytes metabolism, receptor and protein density, as well as oxidative stress.

Advances in computational analyses of multimodal imaging datasets, including deep learning-based applications of artificial intelligence (AI), are just opening the door for a more comprehensive understanding of the pathophysiological cascade of neurodegeneration in motor neuron disease. Here, hypothesis-guided approaches including neuropathological concepts and network-based analyses will be center stage and will eventually find their way into clinical practice.

Crucial information will also be derived from neuroimaging fingerprinting of genetically defined ALS phenotypes like the association with *C9orf72* hexanucleotide repeat expansions, especially in longitudinal investigations of presymptomatic mutation carriers. Neuroimaging offers the possibility to stratify ALS patients according to their intrinsic progression rate, based on cross-sectional and longitudinal studies, thus helping to optimize disease management, enhancing the design of drug trials, and guiding the use of novel individualized treatments when these become available (6, 7).

Further advances in the clinical application of neuroimaging in motor neuron disease will have to rely extensively on a new stage of neuroscientific cooperation, building on existing collaborations between researchers and infrastructures specialized in ALS and facilitating multicenter joint projects that enable grand-scale projects, such as those, for example, led by the Neuroimaging Society in Amyotrophic Lateral Sclerosis (8) or the Canadian ALS Neuroimaging Consortium (CALSNIC) (9). While multicentric studies are most welcome to increase the number of observations in subgroups and different disease stages, the accordingly increasing amount of data collected is paralleled by the need for harmonization of protocols, being the foundation for deducting evidence out of data, and integration of their respective findings into a consistent model of a possible continuing cascade of functional loss.

Integration of multimodal data into a consistent model aiming to correlate the different anatomical, functional, and molecular imaging measurements also calls for the best attempt to perform all measurements to correlate at the shortest intermodality time intervals possible, which is most important in a rapidly progressing disease. But even in the case of a primarily slowly progressing disease, pathological processes can be expected to be accentuated at times, as typically is the case for inflammatory cascades. Any attempt to draw conclusions from observations even a few days apart may thus be jeopardized by the underlying pathological process itself.

The advent of simultaneous PET/MRI, which industries had quickly turned from prototype research instruments into reliable, integrated commercial scanners, has proven to provide a stable, reproducible, and calibrated hybrid modality that not only by design acquires data simultaneously, but also adds value by its potential of dynamically mutually informing their reconstruction algorithms with either modality data. With PET/MRI available at many neuroimaging research sites, the perspective of a one-stop shop for reproducible imaging biomarkers on neuronal damage gains importance, and the call for harmonization of protocols becomes feasible, as the stringent design of hybrid PET/MRI eases the implementation of research protocols and research MR sequences at the existent sites.

The two available PET/MRI scanner models built by the industry providers GE Medical and Siemens Healthineers make use of their respective provider's 3T MRI platform and state-of-the-art PET technology, enabling rapid adaption of advances in image acquisition and reconstruction to their clinical platforms. Improved MR sequences, e.g., providing diffusion imaging at a resolution of 1 × 1 × 1 mm^3^, necessary to characterize microstructural abnormalities (10), can thus be easily adopted and distributed throughout the neuroimaging research sites. Harmonization of MR protocols across different vendors also has proven feasible for the specific needs to characterize microstructural changes in corticospinal, corticorubral, corticostriatal, and hippocampal tracts at different stages of disease progression in ALS (8). The same holds true for protocols for resting state fMRI (11) and MR spectroscopy (MRS) (12).

**Figure 1:**
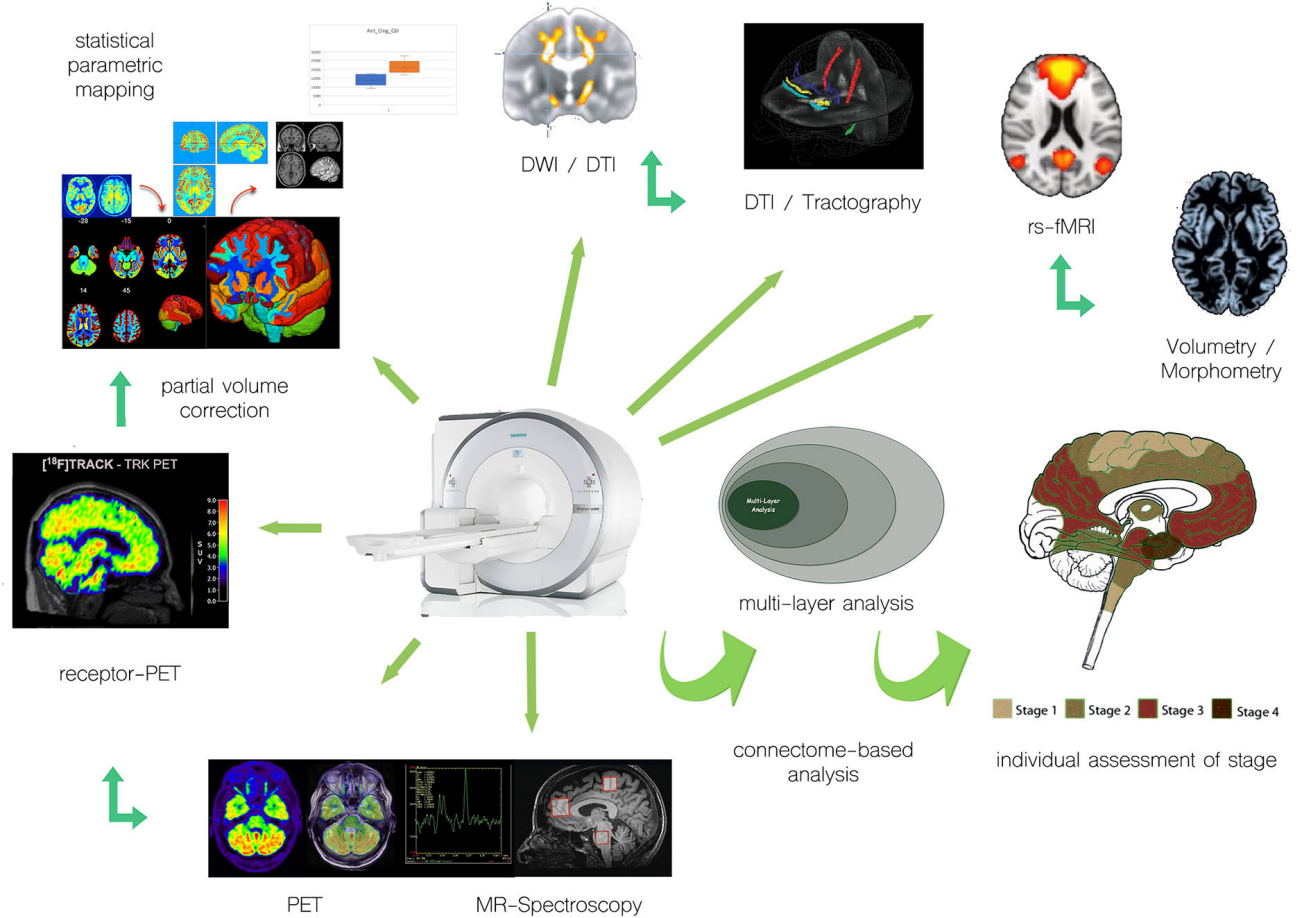
Examples of integration of multimodal PET and MR approaches for individual assessment of motor neuron disease/ALS.

While PET measurements, in principle, allow for absolute quantitation measures, such as regional cerebral blood volume, regional cerebral glucose metabolism, or regional receptor occupancy, the evaluation of multicentric data makes procedures for harmonization of data acquisition and processing mandatory (13–16), which is simplified for PET/MRI, where, by design, variability of the underlying technology is limited. Given that technical prerequisites have been met, quantitative measures of different systems relevant to motor neuron disease can be non-invasively acquired and PET/MRI proves useful for individual assessment of the stage of disease (Figure 1).

## Molecular imaging of disease-inherent pathological alterations—PET

### Glucose metabolism and regional inflammatory changes

While many of the more recent radiotracers are still experimental, measures of regional glucose metabolism using ^18^F-FDG-PET have reached clinical utility in ALS, as evaluated by a panel of experts in the field of nuclear medicine and neurology. By an analysis of the most relevant ^18^F-FDG-PET investigations by the Population, Intervention, Comparison, Outcome (PICO) model, the provided incremental value as compared with the information resulting from the clinical tests routinely performed had been assessed, concluding that ^18^F-FDG-PET offers good evidence to support ALS diagnosis (17). This analysis, however, was based on stand-alone ^18^F-FDG-PET investigations, which did not take into account same-time structural or functional MRI measures. The metabolic patterns identified in ALS consisted of significant hypometabolism in prefrontal, frontal, precentral, and postcentral regions, bilaterally, associated with significant hypermetabolism in posterior occipital and middle temporal cortices, cerebellum, midbrain, and corticospinal tracts, findings which are atypical in functional studies investigating neurodegenerative diseases. Clinically relevant is that the extent of metabolic brain changes in frontal lobes is correlated with cognitive dysfunction (18), thus distinguishing patients with cognitive impairment and possible overlap with frontotemporal dementia from those with pure motor disease (19). The pattern of glucose metabolism also was found to discriminate ALS patients from patients with Parkinson's plus syndromes (20), and, if using machine learning-based techniques, such as support-vector machine discriminant analysis, FDG PET also has proven to be useful for automatically classifying patients with amyotrophic lateral sclerosis vs. controls (21).

More recent studies have found an inverse correlation between precentral and postcentral metabolic activity and clinical stages (22), as well as an inverse correlation between prefrontal and limbic metabolism and apathy (23). When coregistered MRI for partial volume correction was used, FDG PET was able to identify metabolic changes in presymptomatic carriers of the *C9orf72* repeat expansion (24). Also, precentral metabolism distinguished patients with the *SOD1* mutations (SOD-1 ALS) from sporadic ALS (sALS). Specifically, right precentral and paracentral metabolism was relatively increased in patients with SOD-1 ALS as compared to sALS (25). It was hypothesized that a relative increase of ^18^F-FDG-related signal in ALS patients in pyramidal cells in the motor cortex and in their projections to the spinal cord is secondary to a widespread microglial activation and astrocytosis reactive to the reduced neuronal density, with the proliferation of astrocytes being the main determinant of glucose uptake from the intraparenchymal capillaries (26). To better delineate the spatial pattern of metabolic changes in the brain stem and cervical spinal cord, an innovative study capitalized on the potential of integrated PET/MRI to improve result accuracy in small anatomic structures by separately analyzing glucose metabolic patterns in the midbrain/pons, medulla oblongata and cervical spinal cord of ALS and frontotemporal dementia (FTD) patients as compared to normal controls (27). They found a significant and intercorrelating increment in glucose metabolism in the midbrain/pons and medulla oblongata in ALS/FTD patients (spinal-ALS and FTD-motor neuron disease subgroups), interpreted to relate to neuroinflammation, namely activated microglia. While they did not report relevant associations between clinical and metabolic features, medulla oblongata hypermetabolism was associated with shortened survival of ALS patients. In the context of their study, the simultaneously available MRI was instrumental for the identification of corticospinal tract hyperintensities to differentiate ALS from clinically overlapping FTD patients of the motor neuron subgroup, and for the detection of the brain stem and cervical spinal cord hypermetabolism in favor of regional neuro-inflammation linked to activated microglia. Confirmatory studies, directly measuring microglial activation using radioligands targeting TSPO (18 kDa translocator protein), have been performed either using the first-generation TSPO ligand ^11^C-PK-11195 (28, 29) or one of the second-generation ligands ^18^F-FEPPA (30), ^18^F-DPA714 (31), or ^11^C-PBR28, the latter of which has proven to allow for microglia imaging of fiber tracts (32). Technical issues that have not been addressed by a “PET-only” approach for those investigations include the need for partial volume correction, as the measured signal would depend on the regional volume of the anatomical structure of interest. Partial volume correction, however, is extensively dependent on coincident, morphological imaging, where the quality of coregistration of sequentially acquired datasets is a major determinant of bias. It can therefore be expected that using combined information out of a single coordinate system of acquisition, TSPO-PET/MRI will represent an even more useful biomarker in cross-sectionally and longitudinally evaluating the spread of inflammatory lesions.

#### Novel markers for neuroinflammation

##### Purinergic P2X7 ionotropic receptor

More recently, novel markers for neuroinflammation targeting the purinergic P2X7 ionotropic receptor (P2X7R) have been successfully introduced into human PET-imaging [(33) brain kinetic modeling and quantification of brain P2X7 receptors in patients with Parkinson's disease and healthy volunteers] (34). As P2X7R is expressed in astrocytes, microglia, and oligodendrocytes, where they mediate inflammasome signaling (35). A couple of preclinical and clinical studies suggest the implication of P2X7Rs in ALS pathogenesis (36–38). As a whole group of P2X7-Receptor antagonists is currently under development, posing a possible new therapeutic approach for ALS (39). A quantitative assessment of P2X7R as an initial and longitudinal biomarker, along with morphological and functional MR measures will be a basic necessity in according clinical studies, naturally favoring PET/MRI as an imaging modality of choice.

##### C-X-C motif chemokine receptor antagonists

Another promising candidate target suitable as a biomarker for neuroinflammation is the cyclooxygenase-enzyme-2 (COX-2), the possible involvement of which in ALS is indicated by preclinical data (36) and clearly needs systematic *in vivo* studies that will be enabled by the very recent development of appropriate radiopharmaceutical targets (40–42). If a significant involvement of COX-2 could be documented at any stage of disease, this could open the rationale for stage-dependent antioxidative treatment (43, 44), and would again need to be reflected in MRI-based functional measures.

More experimental measures for neuroinflammation are based on findings indicating the implication of C-X-C motif chemokine receptor (CXCR)-4 and CXCR-3, which critically contribute to the disease process in systemic inflammation (45–47), opening another door to possible future therapeutic principles targeting the respective C-X-C motif chemokine receptor (48, 49). As a couple of specific radiopharmaceutical targets have been developed for preclinical and clinical use (40–42, 50), the selection of an appropriate tracer as a biomarker in motor neuron disease would depend on binding characteristics, kinetic analysis, and correlation with functional measures derived from MRI. Again, interpretation of novel preclinical and clinical neuropharmacological imaging data will crucially depend on its supportive correlation with coincident imaging findings using a different modality.

#### Neurotrophins

##### Tyrosine kinase receptor antagonists

There is growing evidence for neurotrophins being involved in neurodegenerative diseases including ALS (40–42, 50, 51). While neurotrophins have not yet proven a significant therapeutic potential in clinical trials, partly because of the difficulties of protein delivery and pharmacokinetics in the nervous system, the binding target of neurotrophins includes a family of tyrosine kinase (TrK) receptors. Within this class of receptors, synthetic antibodies have recently been linked to PET-radioligands targeting the TrK-B receptor, which have passed the preclinical and clinical assessments (52–54). Quantitative characterization of TrK-B alterations in ALS is currently underway by the same research group, accounting for the volumetric changes inherent in the disease by applying PET/MRI in the first place, complemented by magnetic resonance spectroscopy data. The recent development of TrkB agonistic antibodies and BDNF-targeted gene therapies (55, 56) could prove useful, and changes in TrK-B alterations as measured by PET/MRI during targeted therapy could potentially qualify as an imaging endpoint in clinical trials in motor neuron disease (52–54, 57).

#### Markers of neuronal integrity

##### GABA-A—Benzodiazepine receptor complex

The selective PET ligands ^11^C-Flumazenil and 2′-^18^F-Fluoroflumazenil, binding to the GABA-A—receptor, widely expressed on pyramidal neurons, have been suggested to be used as a surrogate *in vivo* marker of neuronal density (58–62). First, applications in neurodegenerative disease have shown a regional neuronal loss in the motor and premotor cortex as well as in extramotor areas in ALS (63, 64), which were also associated with specific cognitive deficits (65, 66). The specificity of GABA-A receptor antagonists to characterize neuronal loss without a possible confound by GABAergic dysfunction has however been questioned, and quantitation methods applied have been scrutinized (67). It has been demonstrated that PET imaging and quantitation using Flumazenil-based radioligands highly depend on the availability of concurrent high-resolution MRI (68), which would ideally be conceptualized by PET/MRI.

##### Antagonists to synaptic vesicle protein 2A

The synaptic vesicle protein 2A (SV2A), a 12-transmembrane domain glycoprotein, is ubiquitously expressed in normal synaptic vesicles throughout the brain, with a particularly high regional expression in the thalamus and basal ganglia (69), only sparing the trigeminal and facial nerve nuclei (70, 71). SV2A has thus been claimed as a potential biomarker for synaptic vesicle density. While it is critical to Ca^2+^-dependent exocytosis (72), its exact physiological role still is subject to further research, and deficiencies in the expression of SV2A have been described in a growing number of neurodegenerative disorders, including frontotemporal dementia (FTD), Parkinson's Disease (PD), Alzheimer's disease (AD), corticobasal degeneration (73–77), as well as further neurological conditions, such as epilepsy (78–80), where it has been identified as the binding site for the antiepileptic drug levetiracetam (81).

In analyses of rat brain homogenates, the number of expressed SV2a proteins per vesicle was found to be highly reproducible at 2–5 copies per vesicle (82, 83). It has been suggested that SV2A plays a central role in exocytosis mediated by Ca^2+^ (71). The deficiency of SV2A in SV2A knockout mice resulted in presynaptic Ca^2+^ accumulation, destabilizing synaptic circuits, and inducing seizures (84). It has also been suggested that SV2A is modulating endocytosis to the SV of the SV protein synaptotagmin-1 (SYT1), and by this mechanism is involved in the homeostasis of the readily releasable pool of SVs (85).

SV2A has thus attracted attention as a target binding site for PET-tracers, and subsequently, with the SV2A antagonist levetiracetam serving as a blueprint, a number of suitable ligands have been developed and translated into human studies (86, 87). The binding of the PET ligand ^11^C-UCB-J to SV2A has been demonstrated to quantitatively correlate and co-localize with synaptophysin (SYN), a key protein located in the pre- or postsynaptic neurons, using a combined *in vivo–in vitro* validation, furthering evidence for SV2A targeting PET tracers to serve as synaptic density marker, which allows for the quantification of synaptic density *in vivo* (88).

Recently, a second-generation SV2A antagonist ^18^F-SynVesT-1 (SDM-8) (89) has been introduced with superior SV2A binding affinity, improved imaging properties, enhanced metabolic stability, and an easier path for radiochemical synthesis (90, 91). For SV2A-markers to prove useful for longitudinal studies of neuronal density in progressive neurodegenerative diseases, such as ALS, confounding effects due to atrophy have to be accounted for, and simultaneous PET/MRI delivers the most accurate, concurrent quantitative anatomic information to perform partial volume correction reflecting the state of morphologic changes at the exact same time point of PET data acquisition.

Further understanding of ALS pathophysiology will be achieved by the study of multimodal MRI and PET data through network-based analyses with hypothesis-guided approaches, including neuropathological concepts, although advanced neuroimaging still awaits translation into clinical settings.

##### Proteinopathy—PET surrogate markers

ALS and the ALS-FTLD spectrum disease are characterized as TDP-43 proteinopathy, where TAR DNA-Binding Protein 43 kDa (TDP-43) links both familial and sporadic forms of ALS. Cytoplasmatic aggregates of TDP-43 are a hallmark of the disease on a cellular level, and protein mislocalization is often regarded as a key mechanism underlying ALS. Up to now, there is no direct PET imaging ligand successfully targeting TDP-43, and out of the notion that multiple pathological proteins may be present in neurodegenerative disease, several groups have investigated the utility of established tau-directed PET tracers to characterize ALS (76, 77, 92–94). As diffusion tensor MR imaging (DTI) has been previously established to identify TDP-43 associated alterations (95), combined PET/MRI will be the modality of choice to further elucidate the role of tau PET imaging in ALS.

### Imaging of disease-inherent pathological alterations—MRI

Neuroimaging with MRI has an essential role in the clinical diagnostic processes for ALS in the exclusion of other etiologies of the clinical presentation (96). In addition, MRI approaches with advanced postprocessing have been established as biological markers of the disease with reliable measures for monitoring disease progression and have greatly improved our understanding of its *in vivo* pathoanatomy (6). Here, diffusion-weighted imaging techniques including diffusion tensor imaging (DTI) had been a focus of research in many studies to investigate microstructural white matter tract alterations in MND (97, 98).

#### Diffusion tensor imaging

By using the DTI, diffusivity in human brain white matter can be non-invasively mapped to first quantify its regional directional dependence, and second, to obtain a reconstruction of fiber tracts by fiber tracking techniques (99). DTI has been established as a robust non-invasive technical tool to investigate the WM neuronal tracts *in vivo* to define anatomical signatures of the different phenotypes of MND and to track *in vivo* the progressive spread of pathological protein aggregates (100). As the neuropathological basis of the ALS-associated propagation patterns in the brain, four neuropathological stages have been defined for ALS, based upon the distribution patterns of phosphorylated 43 kDa TAR DNA-binding protein (101, 102): the sequential protein pathology is spreading initially from the motor neocortex toward the spinal cord and brainstem, followed by spreading to frontal, parietal, and, ultimately, anteromedial temporal lobes. This corticoefferent spreading model has been transferred to DTI-based concepts by a tract of interest (TOI)-based mapping, and DTI seems to be a valid surrogate marker to assess the spreading of TDP-43 pathology *in vivo* within the corresponding neuronal WM tracts (103–105). TOI-based mapping as a hypothesis-driven approach images the neuropathologically proposed sequential progression of ALS in the respective cerebral tract systems, i.e., the CST (as a correlate of ALS-stage 1), the corticorubral and corticopontine tracts (corresponding to ALS stage 2), the corticostriatal pathway (as a correlate of ALS stage 3), and the proximal portion of the perforant path (corresponding to ALS stage 4) (106). This tract-based *in vivo*-staging concept was applied to further ALS variants like primary lateral sclerosis (PLS) (104), lower motor neuron disease/progressive muscular atrophy (107), progressive bulbar palsy (108), and flail limb syndrome (109). In all of these restricted phenotypical ALS variants, an ALS-like *in vivo* alteration pattern of corticoefferent fibers according to the ALS disease propagation model could be shown. DTI-based methods, thus, seem to be a valuable tool for guiding the pathoanatomy definition of MND subtypes, in accordance with current proposals for clinical diagnosis, i.e., the Gold Coast Criteria (110). These results encourage future neuroimaging studies across the phenotypical ALS spectrum to contribute to our understanding of potential modifiers of the clinical presentations in ALS (109).

To this end, it is important to acquire longitudinal imaging data given that longitudinal MRI studies have the potential to provide crucial insights into the natural trajectory of ALS-associated neurodegenerative processes, although it has to be considered that standardized design is required to enable meaningful data interpretation (111). Longitudinal MRI studies in ALS have already been applied to subject groups of heterogeneous sample size (112–116), and reported fractional anisotropy (FA) reduction in the CST as the common core finding. Other DTI measures beyond FA add information on the ALS-associated pathoanatomy, such as a segmental radial diffusivity profile developed by Schuster and colleagues (111). Neurite orientation dispersion and density imaging (NODDI), a multicompartment model of diffusion MRI, demonstrated axonal loss within the CST together with dendritic alterations within the precentral gyrus, suggesting microstructural cortical dendritic changes occur together with CST axonal damage (117).

#### Protocol standardization and multisite MRI data

Advanced imaging protocols with more sophisticated techniques to analyze ever-increasing datasets to guide in the understanding of the anatomical and temporal factors of the biological processes of ALS benefit from collaborations across the entire ALS research community (118). Multicenter approaches like the Neuroimaging Society in Amyotrophic Lateral Sclerosis (NiSALS) with up-to-date, ultimately harmonized neuroimaging protocols aim to obtain high subject numbers and therefore to increase the reliability of results (8, 119, 120). Given that multicenter imaging studies have the limitation of scanner and protocol variability, there were successful approaches to merge data recorded at different sites and/or with different DTI protocols (8, 121, 122).

The Canadian ALS Neuroimaging Consortium (CALSNIC) was established in part to address the challenges associated with protocol variability when pooling multisite data. CALSNIC is a multicenter imaging biomarker validation platform that established from its inception harmonized clinical and imaging protocols across multiple MR platforms. Operating at research sites in Canada and the USA, the platform has conducted two prospective longitudinal studies (CALSNIC1 and CALSNIC2), each including multimodal MRI, neurocognitive assessments, and speech recordings (9). To date, there have been 250 patients with ALS/MND and 200 healthy controls enrolled, with CALSNIC2 ongoing. A recent study of longitudinal DTI-based microstructural alterations in ALS from CALSNIC determined a time interval of about 110 days is the minimum follow-up time to detect longitudinal microstructural alterations (123). Other longitudinal observations within this time frame reported by this consortium include gray and white matter atrophy with deformation-based morphometry (124), altered motor and prefrontal cortex neurochemistry using magnetic resonance spectroscopy (125), and regional texture changes in T1-weighted images (126). Collaborative and multicenter projects like this will be useful in ascertaining the reliability of imaging biomarkers under development (127).

#### MR findings with respect to genetic phenotype

The field of genetic ALS continues to develop rapidly with multiple disease gene discoveries per year (128), with the autosomal dominant inheritance of a hexanucleotide expansion in the first intron of the *C9orf72* gene being the most common cause of familial ALS in people of Northern European ancestry, also as a major contributor to frontotemporal pathology in ALS. DTI studies in patients with *C9orf72* expansion in cross-sectional and longitudinal design demonstrated alterations in motor tracts (129–131); in addition, further white matter areas were found to be affected, e.g., in the frontal white matter (132) and segmentally in the corpus callosum (133). In addition, the *in vivo* histopathological staging approach was also applied to *C9orf72*-associated ALS and demonstrated a corticoefferent involvement pattern according to the staging scheme—a pattern that was not observed in *Super Oxide Dismutase 1*-associated ALS (134). In the last decade, the pre-symptomatic phase of the disease has gained increasing interest, addressing people with family history and genetic risk for ALS without manifestations of the disease (135). Neuroimaging studies in presymptomatic ALS offer opportunities to characterize early genotype-associated signatures and propagation patterns and factors (7). Current initiatives have, thus, integrated natural history and biomarker data on presymptomatic ALS for the design and implementation of pre-symptomatic ALS trials (136). Specifically, in *C9orf72* mutation carriers, DTI studies reported regional reductions of white matter integrity (131, 137), as an indicator of general developmental tardiness. At the spinal level, *C9orf72*-positive subjects older than 40 years were shown to exhibit considerable WM atrophy at C2–C7 vertebral levels in conjunction with progressive pyramidal tract FA reductions (138).

#### Resting-state functional MRI

Brain regions that are co-activated under resting conditions delineate the so-called “resting-state” (RS) functional networks. The assessment of connectivity alterations between RS networks ha**s** provided important insights into brain functional reorganization in several neurodegenerative diseases, including ALS, in which motor and—when present—cognitive impairment may undermine the use of task-based fMRI (139–141).

Several studies showed decreased functional connectivity of the sensorimotor network in ALS patients (142), whereas others found increased connectivity (143), or complex regional patterns of decreased and increased functional connectivity (144, 145). Altered functional connectivity has also been shown in brain networks related to cognition and behavior (especially the default mode and frontoparietal networks) (146, 147), consistent with the multisystem involvement of ALS pathology. It has been suggested that an increase in brain functional connectivity might prevail in earlier stages of the disease as a compensatory mechanism, with a subsequent decrease as pathological burden accumulates. Consistent with this hypothesis, increased functional connectivity was found to be higher in patients with less severe microstructural damage to the CST (148), and associated with a lower rate of disease progression, shorter disease duration (145), and preserved motor function (148). Decreased RS functional connectivity in the sensorimotor and thalamic networks, paralleling progression of structural alterations and clinical decline, was observed over a 2-year period in ALS patients (149). The co-occurrent progressive increase of functional connectivity in extra-motor networks, such as the left fronto-parietal and the temporal RS networks (147, 149), is also consistent with a “disconnection” hypothesis due to the loss of compensation. However, some studies also showed increased functional connectivity within the regions of structural disruption in ALS correlating with faster disease progression (142), and greater clinical and executive cognitive impairment (143, 146). Therefore, a more direct pathogenic involvement of increased functional connectivity related to the loss of local inhibitory circuitry within the primary motor and frontal cortex is also possible.

#### Graph analysis and connectomics

The human brain is a highly integrated neural network consisting of several cortical and subcortical regions that are structurally and functionally interconnected, forming co-operating sub-networks. Graph theoretical models have conceptualized such complex organization as the brain “connectome”, consisting of anatomic regions defined as “nodes”, which are linked by “edges” (i.e., structural or functional connections). In ALS, graph analysis and connectomics might represent a powerful approach to detect upper motor neuron degeneration, extramotor brain changes, and network reorganization associated with the disease.

Two independent studies applied network-based statistics to DT MRI of patients with ALS, both demonstrating the presence of an impaired sub-network including bilateral primary motor regions, supplementary motor areas, basal ganglia, and associative parietal areas (150, 151). Patients with a *C9orf72* mutation showed a more widespread white matter involvement (152). In a longitudinal study, the sub-network of impaired connectivity expanded over time, involving frontal, temporal, and parietal regions (150), consistent with the proposed model of TDP-43 pathological spreading. In line with such hypothesis, a study evaluated brain structural connectivity in a consistent set of healthy controls, showing that regions involved in subsequent stages of ALS pathology are highly interconnected by WM tracts, which may serve as anatomical “infrastructures” facilitating TDP-43 spread (153). More recently, a computational model was applied to the MRI scan of ALS patients to simulate this progressive network degeneration (154). Computer-simulated aggregation levels mimic true disease patterns in ALS patients. Simulated patterns of involvement across cortical areas show significant overlap with the patterns of empirically impaired brain regions on later scans, in accordance with established pathological staging systems (152).

Few studies applied network-based analyses to the assessment of functional alterations in ALS patients using resting-state functional MRI (rs-fMRI), demonstrating complex connectivity alterations encompassing frontal, temporal, and occipital regions (155, 156). A recent study assessed the functional and structural connectivity patterns across the ALS-FTD spectrum, investigating whether and where MRI connectivity alterations of ALS patients with any degree of cognitive impairment (i.e., ALS-ci/bi and ALS-FTD) resembled more the pattern of connectome damage of ALS or bvFTD (157). As compared with controls, ALS-ci/bi patients demonstrated an “ALS-like” pattern of structural damage, diverging from ALS without cognitive impairment with similar motor impairment for the presence of enhanced functional connectivity within sensorimotor areas and decreased functional connectivity within the “bvFTD-like” pattern. On the other hand, ALS-FTD patients resembled both structurally and functionally the bvFTD-like pattern of connectome damage with, in addition, the structural ALS-like damage in the motor areas. A maladaptive role of functional rearrangements in ALS-ci/bi concomitantly with similar structural alterations compared to ALS without cognitive impairment supports the hypothesis that ALS-ci/bi might be considered as a phenotypic variant of ALS, rather than a consequence of disease worsening.

In a multicenter study (158), compared with healthy controls, patients with ALS and patients with PLS showed altered structural global network properties, as well as local topologic alterations and decreased structural connectivity in sensorimotor, basal ganglia, frontal, and parietal areas. Patients with PMA showed, instead, preserved global structure. Increased local functional connectivity was observed in patients with ALS in the precentral, middle, and superior frontal areas, and in patients with PLS in the sensorimotor, basal ganglia, and temporal networks. In patients with ALS and patients with PLS, structural connectivity alterations correlated with motor impairment, whereas functional connectivity disruption was closely related to executive dysfunction and behavioral disturbances (158).

#### Magnetic resonance spectroscopy

In addition to evaluating structural, microstructural, and functional changes, magnetic resonance spectroscopy (MRS) permits the probing of neurochemical correlates of neurodegeneration in ALS (12). Numerous studies using varying techniques (single voxel or multivoxel) have consistently revealed reduced N-acetylaspartate (NAA, a chemical marker of neuronal integrity) in motor and extra-motor regions in ALS. Other metabolites of interest include myo-inositol (mIns, a glial marker), and the excitatory and inhibitory neurotransmitter system involving glutamate, glutamine, and GABA (159). Technological advances in MR hardware and spectral acquisition and editing methods have increased the ability to more readily resolve such metabolites. For example, this includes ultra-high field studies at 7 tesla for glutamate, glutamine, and GABA (160, 161) and MEGA-PRESS for GABA (162, 163) detection. Combined PET-MR imaging with MRS provides the opportunity to explore complementary pathological or pathophysiological mechanisms simultaneously from the molecular and neurochemical perspectives. In a PET-MR study in ALS that included MRS motor cortex inflammation, measured using the TSPO tracer [^11^C]-PBR28, and gliosis, measured using the myo-inositol signal, were found to be directly correlated in the motor cortex (164).

#### Machine learning classifiers

One of the overarching aims in advanced neuroimaging biomarker development in neurodegenerative disorders like ALS is the observer-independent classification of imaging data for individual patient's stratification for later use in multicenter therapeutic trials; to this end, there is a rapidly growing interest in Machine Learning (ML) models, classifiers, and predictive modeling in ALS (165). The choice of the ML model in ALS neuroimaging needs to be carefully tailored to a proposed application based on the characteristics of the available data and the profile of the candidate model, as proposed by Grollemund et al. (166). A recent systematic review on MRI feature selection for ML-based neuroimaging classifiers in ALS suggested the integration of DTI, volumetric, and texture data (167), but potential future applications might include a multiparametric MRI combination of more approaches such as intrinsic functional connectivity MRI. Connectome-based analyses of multiparametric MRI have already demonstrated their potential as a tool for patient stratification and as a prognostic biomarker in ALS to predict disease progression (154). PET/MRI is able to provide a multiparametric protocol, where a multimodal composite score may combine the aforementioned PET and MR techniques to address specific questions [e.g., (168)]—for a review see (169).

## Summary

Neuroimaging fingerprinting of genetically defined ALS phenotypes will be important, especially longitudinal investigations of presymptomatic mutation carriers. Neuroimaging offers the possibility to undertake cross-sectional and longitudinal studies to stratify ALS patients according to their intrinsic progression rate, thus helping to optimize disease management, enhancing the design of drug trials, and guiding the use of individualized treatments when these become available. Recent research has contributed to the change in perception of neuroimaging in motor neuron disease, which traditionally had been primarily an academic tool with limited direct relevance to individualized patient care, but, with the advances in computational imaging, has emerged as a viable clinical tool with true biomarker potential (170).

MRI and PET provide methodologically different and partially complementary information on disease pathology. There are multiple aspects where applied neuroimaging and biomarker imaging strategies in neurodegenerative disease are influenced by and directly benefit from simultaneous PET/MRI:

- Concurrent acquisition and analysis augment the precision of partial volume correction for PET data by minimizing the main confounds introduced by small misregistration and data resampling inaccuracies of *post-hoc* coregistration of PET and segmented MRI acquired at different time points and in different scanner coordinate systems (171, 172).
- Novel regularized PET image reconstruction techniques based on anatomical priors derived from concurrent MRI significantly improve PET image quality (173–175).
- Novel readout and quantitation techniques including radiomics and machine learning/artificial intelligence informed algorithms benefit directly from inherently coregistered data and the high degree of standardization possible in PET/MRI, likely to result in improved performance of AI applications (176– 180).
- Data consistency of simultaneous PET/MRI improves data pooling of different varieties of radioligands of the same functional target, assisting in moving toward multicenter therapeutic trials.
- Simultaneous PET/MRI and the inherent temporal synchronicity of findings will be instrumental in the development of tailored imaging probes or assessing the effects of drug challenges in treatments (181–184).
- PET/MRI enables to the design of more complex prospective trials using multiple tracers to characterize a disease, capitalizing on an intelligent spread of complex MR protocols over consecutive PET/MRI sessions using different tracers to max out the gain of information by each session and still ensure patient compliance (185).

Future developments may include a possible combination of rapid multi-tracer PET in a single PET/MRI session, making use of the high spatial information provided by MRI to improve signal separation in multi-tracer and multi-isotope studies, where typically staggered injection of ultra-short lived radionuclides combined with longer half-lived ones is practiced, and where spatial registration between different stages is crucial (186). Other future applications might include the combination of simultaneous PET/MRI with hyperpolarized MR imaging (187), to add even more layers of complementary metabolic information.

To summarize, the roles of MRI and PET as straight-forward diagnostic tools in ALS and further neurodegenerative disorders are emerging; the concepts to use them as a biological marker or as a read-out in clinical trials are existing and have to be probed for their clinical relevance. Combined PET/MRI has the potential as a future gold standard for characterizing motor-neuron disease and offers an important contribution to the standardization of imaging across multiple centers.

## Author contributions

FJ, JK, and H-PM contributed to conception and design. FJ wrote the first draft of the manuscript and designed the figure and done the final revision of the manuscript. JK, H-PM, SK, and FA contributed complete sections. FW, AT, WT, and RS performed a review for intellectual and scientific content. All authors contributed to the article and approved the submitted version.

## Conflict of interest

FJ serves as Associate Editor for Frontiers in Neurology, Section Applied Neuroimaging and as Associate Editor for Frontiers in Oncology, Section Cancer Imaging and Image-directed Interventions. He has received the ALS Canada-Brain Canada Discovery Grant 2021 and receives or has received research operating funds from Biogen and the University of Alberta. SK has received research operating funds from Biogen. FA serves as Section Editor of NeuroImage: Clinical. She has received speaker honoraria from Roche and Biogen Idec; and receives or has received research supports from the Italian Ministry of Health, AriSLA (Fondazione Italiana di Ricerca per la SLA), the European Research Council and Foundation Research on Alzheimer Disease. H-PM serves as Associate Editor for Frontiers in Neurology, Section Applied Neuroimaging. JK serves as Specialty Chief Editor for Frontiers in Neurology, Section Applied Neuroimaging and as Associate Editor Neurology for Therapeutic Advances in Chronic Disease. He has received consulting fees as an advisory board member or honoraria as a speaker from AbbVie, BIAL, Biogen, Boehringer Ingelheim, Desitin, Esteve, Licher MT, Medtronic, Novartis, STADA, UCB Pharma, Zambon. The remaining authors declare that the research was conducted in the absence of any commercial or financial relationships that could be construed as a potential conflict of interest.

## Publisher's note

All claims expressed in this article are solely those of the authors and do not necessarily represent those of their affiliated organizations, or those of the publisher, the editors and the reviewers. Any product that may be evaluated in this article, or claim that may be made by its manufacturer, is not guaranteed or endorsed by the publisher.
